# Visible-Light-Driven Ag-Doped BiOBr Nanoplates with an Enhanced Photocatalytic Performance for the Degradation of Bisphenol A

**DOI:** 10.3390/nano12111909

**Published:** 2022-06-02

**Authors:** Chu-Ya Wang, Qi Zeng, Li-Xia Wang, Xin Fang, Guangcan Zhu

**Affiliations:** School of Energy and Environment, Southeast University, Nanjing 210096, China; zeng_qi@seu.edu.cn (Q.Z.); 220210571@seu.edu.cn (L.-X.W.); fxin@seu.edu.cn (X.F.)

**Keywords:** bismuth oxybromide (BiOBr), Ag-doping modification, visible light, charge separation, bisphenol A (BPA)

## Abstract

Based on the low utilization rate of visible light and the high-charge carriers-recombination efficiency of bismuth oxybromide (BiOBr), in this work, noble metal Ag was used to modify BiOBr, and Ag-doped BiOBr nanoplates (Ag-BiOBr) were obtained through a one-step hydrothermal method. Compared with BiOBr, the absorption edge of Ag-BiOBr showed a redshift from 453 nm to 510 nm, and the absorption efficiency of visible light was, obviously, improved. Bisphenol A (BPA) was chosen as the target pollutant, to evaluate the photocatalytic performance of the samples. Ag_0.1_-BiOBr showed the highest degradation efficiency. The intrinsic photocatalytic activity of Ag_0.1_-BiOBr, under visible light, was approximately twice as high as that of BiOBr. In this way, a new visible-light-driven photocatalyst was proposed, to fight against organic pollution, which provides a promising strategy for water and wastewater treatment.

## 1. Introduction

Numerous organic pollutants have been discharged into the aqueous environment, causing series of environmental pollution problems in the past decades. The impact of these organic pollutants on production and life is growing. Many kinds of organic pollutants, such as endocrine disruptors, antibiotics and other emerging contaminants, cannot be effectively removed through traditional treatment methods [1], and, thus, attract extensive attention. Since the concept of photocatalysis was put forward in the 1970s, this kind of technology, with no secondary pollution, low energy consumption and high efficiency, has been considered as an ideal strategy to deal with organic contaminants [2,3].

Among all kinds of photocatalysts, Bismuth oxybromide (BiOBr) has a high carrier separation efficiency, due to its special layered structure. The [Bi_2_O_2_]^2+^ layer is arranged alternately with the [Br_2_]^2–^ layer, and, usually, the [Bi_2_O_2_]^2+^ layer is sandwiched between two [Br_2_]^2–^ layers [4,5]. This special open-layered structure provides enough space for polarized atomic orbitals, inducing an internal electric field perpendicular to the atomic layer [6]. It promotes the separation of photogenerated electrons (e^–^) and holes (h^+^) [7], exhibiting favorable photocatalytic activity [8]. In addition, BiOBr is an indirect bandgap semiconductor, and the e^–^ need to cross the K space to reach the conduction band (CB), which can suppress the recombination rate of photogenerated e^–^ and h^+^ [9]. However, the separation efficiency of photogenerated e^–^ and h^+^ still needs to be improved for practical application. Moreover, the low response to visible-light irradiation is another limitation of BiOBr for its photocatalytic performance. 

To improve the photocatalytic activity of BiOBr, many efforts has been made recently, including metal doping, ion doping and heterostructure construction [10,11,12]. For example, AgBr-Ag-BiOBr photocatalyst with the p-metal-n structure exhibited a superior performance concerning degradation of methyl orange under visible-light irradiation (λ > 420 nm) [13]. Ag/AgBr/Ga_2_O_3_ composite with heterojunction microstructure was synthesized, by using chemical deposition, and the degradation efficiency of methyl orange within 30 min was 89% [14]. The colored dyes with dye sensitization were chosen in both two works, which could, also, be highly degraded using advanced oxidation processes, such as subcritical and supercritical water oxidation [15,16]. Except for the construction of heterojunction, many studies have shown that noble doping is an effective way to tune the photocatalytic properties of the material. For example, TiO_2_ doped with Ag shows a better photocatalytic performance under visible-light irradiation because the doped Ag element act as a capture center and facilitate the separation of photogenerated e^–^ and h^+^ [17]. In addition, Ag-doped ZnO has a wider light response range than that of ZnO [18]. These results indicate that Ag-doping modification can effectively promote the photocatalytic activity of the semiconductor photocatalyst. Inspired by these previous works, Ag-doping modification might be favorable, to enhance the response to visible light as well as improve the charge separation and transfer efficiency. Compared with the heterostructure construction, Ag doping means that catalysts possessed a smooth surface, and Ag elements displayed a high dispersion in crystal of catalysts, with the form of Ag(I) instead of Ag(0).

In this work, Ag-doped BiOBr (Ag-BiOBr) were prepared through a hydrothermal process. The comparison, with already published work, was shown in Table S1. A series of characterizations had been carried out, systematically, to compare the changes of the morphology, band structure and electrochemical properties, before and after Ag-doping modification. To avoid the dye-sensitization process, bisphenol A (BPA), one kind of colorless pollutants, was chosen to evaluate the photocatalytic activity of the samples. Then, capture experiments of free radicals were performed to explore the reaction mechanism of photocatalytic degradation, thus elucidating the relationship between structure and photocatalytic activities.

## 2. Materials and Methods

### 2.1. Synthesis of Ag-BiOBr Photocatalysts

Bismuth nitrate pentahydrate (Bi(NO_3_)_3_·5H_2_O), ethylene glycol (EG), Ammonium bromide (NH_4_Br), distilled water and silver nitrate (AgNO_3_). All chemicals used in this work were of analytical grade. EG and AgNO_3_ were purchased from Sinopharm Chemical Reagent Co., Ltd. (Shanghai, China), and others were obtained from Aladdin Reagent Co., Ltd. (Shanghai, China). They were used, directly, without any further purification. In a typical procedure, 0.485 g of Bi(NO_3_)_3_·5H_2_O (1 mmol) was added into 5 mL EG. After 5 min continuous ultrasonic treatment and stirring, a homogeneous dispersion was obtained. Meanwhile, 0.103 g of NH_4_Br (1 mmol) was completely dissolved into 30 mL of distilled water. After that, these two aforementioned solutions were mixed with continuous stirring for 4 min and an obvious suspension was obtained, immediately. At same time, different amounts of AgNO_3_ (0.05, 0.1 and 0.2 mmol) were added, and the products were noted as Ag_0.05_-BiOBr, Ag_0.1_-BiOBr and Ag_0.2_-BiOBr, respectively. Then, the mixture was transferred into a 50 mL autoclave with Teflon linear, which was heated at 160 °C for 12 h. After cooling to ambient temperature, naturally, the product powders were collected through centrifugation and the samples were washed with distilled water and alcohol for three times, respectively, to remove the residuals and organics. Finally, the samples were dried in vacuum at 80 °C for 10 h. In addition, BiOBr sample was fabricated through the same process with the absence of AgNO_3_.

### 2.2. Characterizations

The crystallinity of samples was detected using an X-ray diffraction (XRD) diffractometer (Bruker D8 Advance, Karlsruhe, Germany), equipped with a mono Cu Kα (λ = 1.541874 Å). And the version of measurement software was V6.5.0 (32 Bit) (Bruker AXS, Karlsruhe, Germany). Moreover, a UV-vis-NIR spectrometer (UV-3600, Shimadzu, Kyoto, Japan) was used to measure diffuse reflectance spectra (DRS) of the samples. The X-ray photoelectron spectroscopy (XPS; Nexsa, ThermoFisher, Waltham, MA, USA) was used to determine the chemical compositions and the valence potential of the as-prepared samples. The morphologies of the samples were measured using scanning electron microscopy (SEM; JSM-700F, JEOL, Akishima, Japan), transmission electron microscopy (TEM; FEI Talos F200s, ThermoFisher, Waltham, MA, USA) and high-resolution TEM (HRTEM; FEI Talos F200s, ThermoFisher, Waltham, MA, USA). The elements were confirmed through energy dispersive spectrometer (EDS; FEI Talos F200s, USA). The existence of free radicals was tested through an electron paramagnetic resonance (EPR) spectrometer (Bruker A300, Bruker, Munich, Germany)

### 2.3. Electrochemical Measurements

All electrochemical characterizations were carried out on the CHI760E electrochemical workstation (CH Instrument Co., Shanghai, China) using a three-electrode system. The Pt wire and the Ag/AgCl (KCl, 3 M) were used as the counter electrode and the reference electrode. Quartz glass was used in photocurrent experiment, and the other parts were ordinary glass products. The working electrodes used in the electrochemical impedance spectroscopy (EIS) measurements, Mott–Schottky plots texts and photocurrent responses tests were prepared as follows: 5 mg catalyst was ultrasonically dispersed in 1 mL methanol, then 10 μL nafion solution was added to the mix well and dripped onto the glassy carbon electrode and F-doped SnO_2_ (FTO) glass, respectively. EIS tests were performed in 0.05 M K_3_[Fe(CN)_6_] and K_4_[Fe(CN)_6_] electrolyte solution at an alternating current frequency of 1~10^6^ Hz and a voltage amplitude of 5 mV. Mott–Schottky plots texts were performed in 0.1 M Na_2_SO_4_ electrolyte solution at a frequency of 1000 Hz and an alternating current voltage amplitude of 5 mV. Photocurrent tests were performed in 0.1 M Na_2_SO_4_ electrolyte solution with a bias voltage of 0.3 V.

### 2.4. Photocatalytic Activity Evaluation

The photocatalytic degradation activity of the samples was measured using a 500 W Xenon lamp (CHF-XM500, PerfectLight, Beijing, China) with a 420 nm cutoff filter at room temperature. The target pollutant was 10 mg/L of BPA solution. In a typical degradation process for each experiment, 10 mg of photocatalyst powder were added into 50 mL of the aforementioned BPA solution, and the mixture was kept being stirred for 30 min, in order to achieve the adsorption–desorption equilibrium. At a specific time interval, 0.5 mL of solution was taken out from the reaction system and immediately centrifuged. After that, BPA concentrations of the obtained samples were measured using a high-performance liquid chromatography (HPLC, Primaide, Hitachi, Dalian, China). The temperature of the chromatographic column was 30 °C. The mobile phase was composed of deionized water, acetonitrile and formic acid with a volume ratio of 1000:1000:1, and the flow rate was set as 0.5 mL/min. All experiments were conducted twice. The intermediate compounds of BPA degradation were determined through Liquid Chromatograph Mass Spectrometer (LC-MS; LC-MS-2020, Shimadzu, Kyoto, Japan). The preliminary preparation of catalyst cyclic stability test was the same as the above. Centrifugation was performed after each degradation, and the obtained catalyst was used for the next degradation test, with a total of 5 cycles.

## 3. Results and Discussion

### 3.1. Characterizations

**Figure 1:**
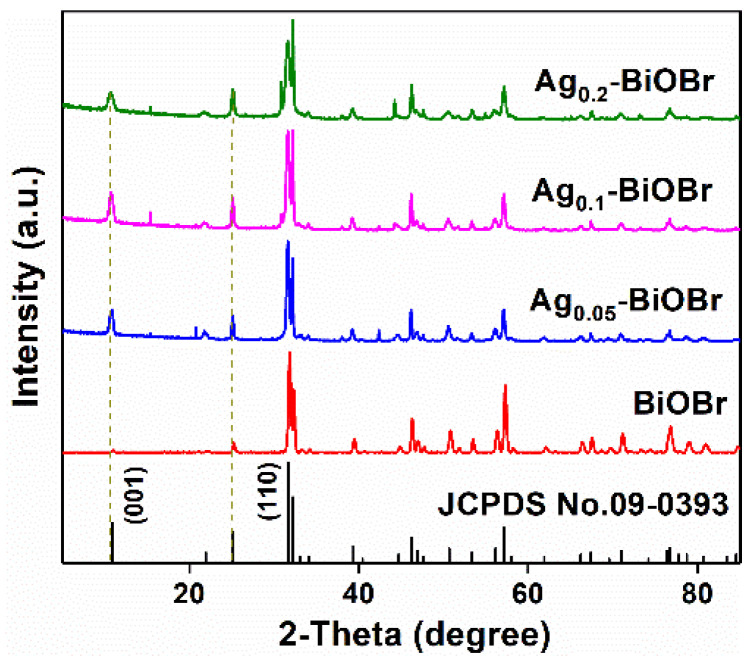
XRD patterns of BiOBr and Ag-BiOBr.

The phase composition of the samples was determined using XRD. The diffraction peaks in the XRD spectrum (Figure 1) could be indexed to BiOBr (JCPDS No. 09-0393). There are no impurity peaks in these peaks, and the diffraction peaks are sharp, indicating that the crystallinity and purity were high [19]. The diffraction peak of Ag-BiOBr is consistent with that of BiOBr from the spectrum, which indicates that Ag-doping modification did not affect the phase of BiOBr frame as the host crystal. However, no obvious signals of Ag are obtained, indicating that Ag element might be highly dispersed in the BiOBr catalyst. It is worth noting that the peak at 10.9°, which represents the (001) plane, was substantially lower than that of the (110) plane at 32.2°, indicating that the crystal grew along the direction of [110]. In short, the (001) plane of these materials was highly exposed and the (110) plane was suppressed. Moreover, as is shown in Figure 1, according to Braggs law, the position of the peak slightly shifted to low angle after Ag-doping modification, indicating that Ag doping increased the crystal cell parameters of the sample accordingly [20]. The expansion of the lattice may be attributed to the large ionic radius of Ag^+^ (1.26 Å) than Bi^3+^ (1.08 Å).

**Figure 2:**
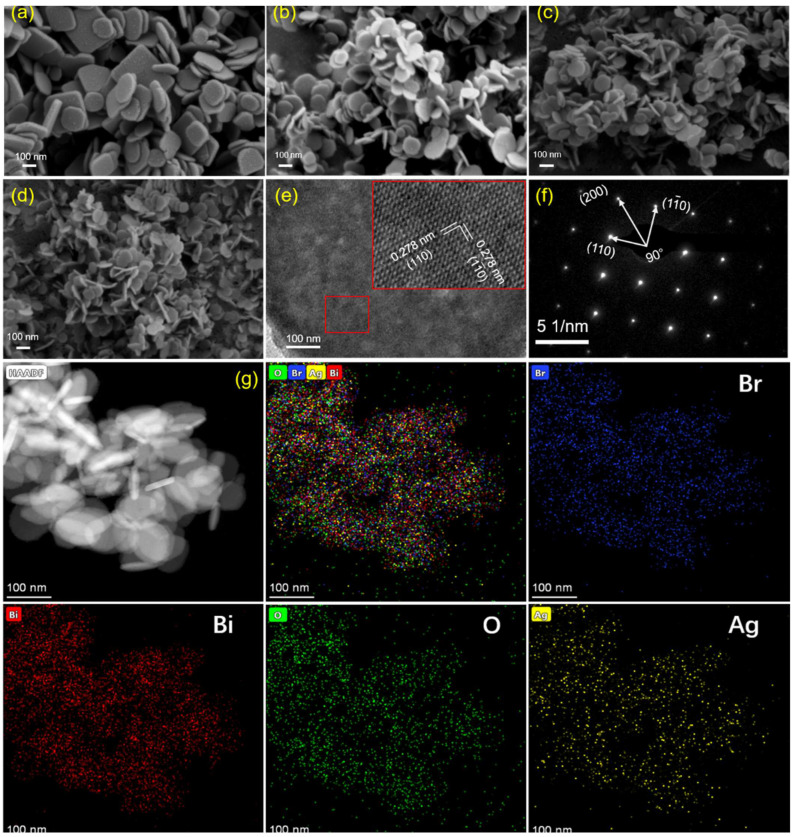
SEM of (**a**) BiOBr, (**b**) Ag_0.05_-BiOBr, (**c**) Ag_0.1_-BiOBr, (**d**) Ag_0.2_-BiOBr, (**e**) HRTEM, (**f**) SAED, (**g**) STEM and corresponding EDS mapping images of Ag_0.1_-BiOBr.

The morphologies of the samples were observed using SEM. As is shown in Figure 2a–d, both BiOBr and Ag-BiOBr were two-dimensional nanoplate structures, and no other impurities were observed, indicating that the products had a high purity. Moreover, Ag-doped products were decreased on the spatial scale of nanoplates. However, the diameters and thicknesses of catalysts, with different amount of the doped Ag element, are similar. In addition, the smooth surface, also, indicates that there was no Schottky junction and heterojunction formation on the BiOBr surface, indicating the high uniformity of the nanoflake. After Ag-doping modification, the mean size of Ag_0.1_-BiOBr nanoplates was 50–80 nm, and the thickness was about 30–50 nm. HRTEM image shows a clear and continuous lattice spacing (0.278 nm) (Figure 2e). These lattice spacings can be assigned to the (110) and (1–10) planes of tetragonal BiOBr [21,22], and no imperfect point was observed. This further confirms that the doped Ag is well incorporated in the host BiOBr lattice, which was further confirmed by the result of SAED in Figure 2f. EDS mapping was used to confirm the Ag presence in BiOBr (Figure 2g). EDS mapping images display the elemental composition of Ag_0.1_-BiOBr, and it revealed the homogeneous distribution of Br, Bi, O and Ag elements in the selected areas, indicating the successful of Ag-doped BiOBr nanoplates. 

**Figure 3:**
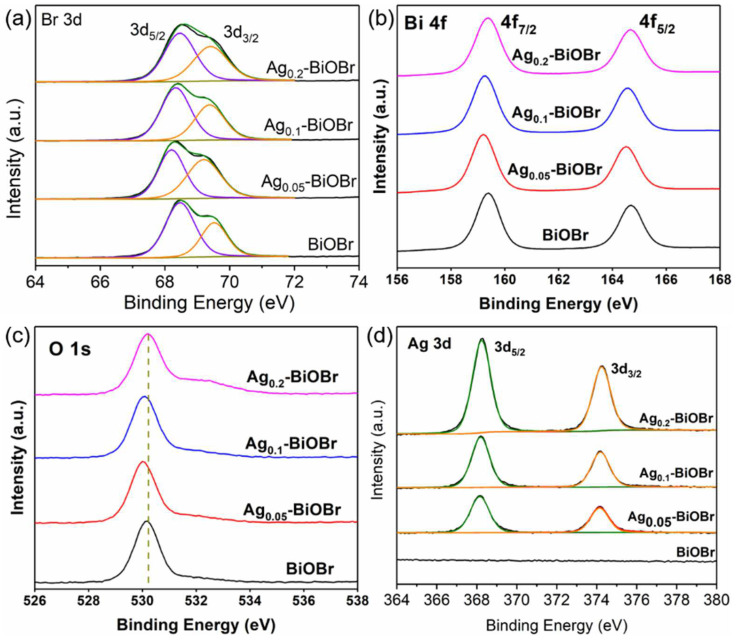
(**a**) Br 3d, (**b**) Bi 4f, (**c**) O 1s and (**d**) Ag 3d of BiOBr and Ag-BiOBr.

XPS tests were, also, conducted to verify the elemental composition of the catalysts. According to the survey spectrum (Figure S1), the elemental compositions of the samples before and after Ag-doping modification were similar, and both contained Bi, O and Br elements. Moreover, it can be found that the survey spectrum of Ag-BiOBr samples possesses a diffraction peaks of Ag 3d near 400 eV. As is shown in Figure 3a, the Br 3d spectrum shows two peaks at 68.2 eV and 69.2 eV, with a splitting energy of 1.0 eV, corresponding to Br 3d_5/2_ and Br 3d_3/2_ [23], respectively. The two peaks at 159.2 eV and 164.6 eV (splitting energy of 5.4 eV), represented by Bi 4f_7/2_ and Bi 4f_5/2_ [24,25] (Figure 3b). In the O 1s spectrum (Figure 3c), a peak at 530.28 eV corresponded to lattice oxygen. In Figure 3d, two peaks at 368 eV and 374 eV with a splitting energy of 6.0 eV belong to 3d_5/2_ and 3d_3/2_ of Ag(I) [26,27], respectively. The peak intensity of Ag 3d was enhanced with its doping concentration. Compared with BiOBr, Ag was successfully doped into the BiOBr crystal, and it shows that Ag did not exist in the form of Ag(0) but in the form of Ag(I) in the BiOBr framework. Ag(I) increased the electron densities of other elements, and, thus, led to the shift of XPS signals towards lower binding energy.

### 3.2. Band Structures and Electrochemical Properties

**Figure 4:**
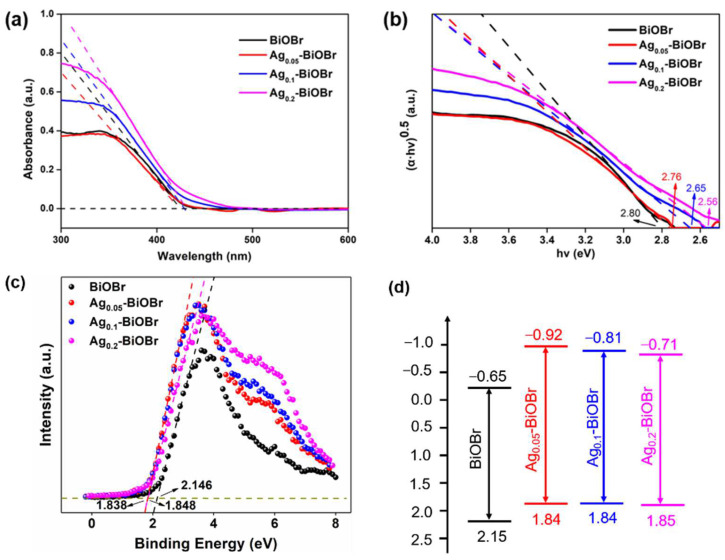
(**a**) UV-vis DRS, (**b**) Tauc plots, (**c**) valence band and (**d**) band structure diagrams of BiOBr and Ag-BiOBr.

The photocatalytic activity was greatly limited by the light absorption property, which highly relies on the band structure of semiconductor. Therefore, UV-vis diffuse reflectance (UV-vis DRS) was performed, to identify the absorption edges of the as-prepared photocatalysts. In Figure 4a, the edges of Ag_0.05_-BiOBr, Ag_0.1_-BiOBr and Ag_0.2_-BiOBr red-shifted to 425 nm, 430 nm and 432 nm, respectively, indicating that the Ag-doping modification can enhance the visible-light response to some extent. Namely, the doped Ag element could adjust the band structure of BiOBr and broaden the light response range of catalyst. However, Ag_0.2_-BiOBr sample coincided with the original BiOBr absorption edge data, indicating that the widening effect would be reduced with the increase in Ag addition. In Figure 4b, band gaps of BiOBr, Ag_0.05_-BiOBr, Ag_0.1_-BiOBr and Ag_0.2_-BiOBr were calculated on the basis of Tauc plots [28], which were calculated using the following equation [29]:

(1) $$\alpha hv=A{\left(hv-E_{g}\right)}^{n/2}$$

where α, hv, A and $E_{g}$ are the absorption coefficient, photon energy, a constant and the band gap, respectively. The *n* value is 4 for BiOBr, as a typical indirect band gap semiconductor. The results of band gaps were calculated as 2.80 eV, 2.76 eV, 2.65 eV and 2.56 eV, respectively, confirming that the doped Ag element could tailor the band structure and reduce the band gap of BiOBr. Moreover, excessive Ag doping destroyed the order of the internal lattice of BiOBr, resulting in more defects in the framework, and the charge carriers were captured halfway rather than transferred to the catalysts surface. As is shown in Figure 4c, the valence band (VB) position was determined using VB spectrum. The VB top potential of BiOBr was 2.15 eV, and both of Ag_0.1_-BiOBr and Ag_0.05_-BiOBr were 1.84 eV, while that of Ag_0.2_-BiOBr was 1.85 eV. Ag-doping modification made the VB top less positive, changed band structure and weakened the oxidizability of h^+^. However, the CB bottom potential of Ag_0.1_-BiOBr and Ag_0.2_-BiOBr were more negative than that of BiOBr, indicating that photogenerated e^–^ reducibility was enhanced after Ag-doping modification. Moreover, the band structure of the samples could be obtained according to the value of band gap and VB top potential (Figure 4d). 

**Figure 5:**
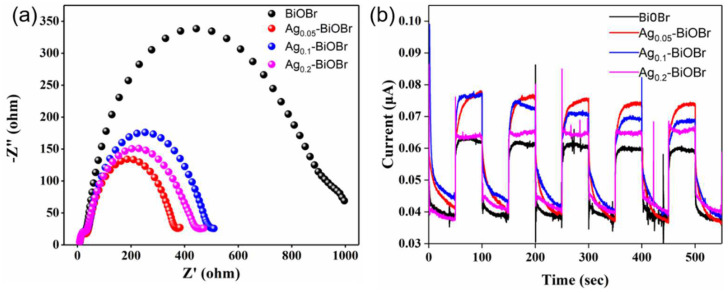
(**a**) EIS spectrum and (**b**) photocurrent response test plots of BiOBr and Ag-BiOBr.

The separation and transfer efficiency of photogenerated e^–^ and h^+^ were measured through EIS tests. In Figure 5a, the curvature radius of the curves decreased, obviously, after Ag-doping modification, indicating that the doped Ag element could significantly reduce the electrochemical impedance of BiOBr, which facilitate the separation and transfer of charge carries. Moreover, transient photocurrent response tests (Figure 5b) were performed to further characterize charge separation and transfer properties. The photocurrent response intensity of these samples had the following order: Ag_0.05_-BiOBr > Ag_0.1_-BiOBr > Ag_0.2_-BiOBr > BiOBr. The photocurrent response intensities of Ag_0.05_-BiOBr, Ag_0.1_-BiOBr and Ag_0.2_-BiOBr were 1.23, 1.16 and 1.05 times higher than that of BiOBr, respectively. The results show that that the Ag-BiOBr nanosheets possessed better electrochemical properties compared with BiOBr.

### 3.3. Photocatalytic Degradation of BPA

**Figure 6:**
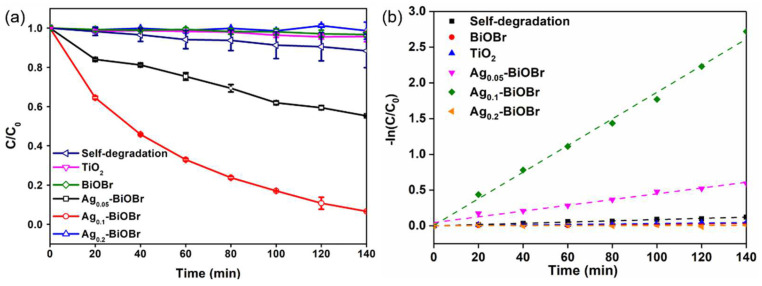
(**a**) Photocatalytic degradation curves and (**b**) corresponding kinetic curves of the BiOBr and Ag-BiOBr samples.

In order to investigate the photocatalytic performance of the prepared catalysts under visible-light irradiation (λ ≥ 420 nm), the BPA degradation efficiency over different photocatalysts under a 140 min irradiation was tested. In Figure 6a, the result of 30 min dark treatment before the reaction confirmed that the adsorption amount of BPA on the catalyst was very limited. In addition, the self-degradation of BPA without any photocatalyst under visible-light irradiation was negligible. Under the 140 min visible-light irradiation, the degradation rates of BPA over BiOBr, Ag_0.05_-BiOBr, Ag_0.1_-BiOBr and Ag_0.2_-BiOBr reached 55.9%, 81.8%, 75.2% and 62.1%, respectively. As aforementioned, the absorption edges of Ag-BiOBr were red-shifted, to 462 nm from 453 nm, after Ag-doping modification, which improved the visible-light absorption of Ag-BiOBr. 

To quantitatively compare the photocatalytic activity of these catalysts, the pseudo-first-order kinetic equation was used to fit BPA degradation data:

ln(*C_t_*/*C*_0_) = *kt* (2)

where *C_t_*, *C*_0_, *k* and *t* are the concentration of BPA at each reaction time, initial concentration, kinetic constant and irradiation time, respectively. The first-order kinetic fittings of photocatalytic degradation of BPA by these samples are shown in Figure 6b. The calculated *k* values were 0.002 min^−1^, 0.004 min^−1^, 0.018 min^−1^ and 0.001 min^−1^ for BiOBr, Ag_0.05_-BiOBr, Ag_0.1_-BiOBr and Ag_0.2_-BiOBr, respectively. Considering that the specific surface area of the catalyst was related to the exposed active site and the light visible area of the catalysts, thus affecting the catalytic performance, BET surface areas (*S*_Bet_) of these samples were tested. The values of *S*_Bet_ were 12.275 m^2^∙g^−1^, 15.438 m^2^∙g^−1^, 15.202 m^2^∙g^−1^ and 20.549 m^2^∙g^−1^ for BiOBr, Ag_0.05_-BiOBr, Ag_0.1_-BiOBr and Ag_0.2_-BiOBr (Table S2), respectively. The photocatalytic performance of each sample was calculated after eliminating the influence of specific surface area, which represented the size of catalytic reaction performance per unit specific surface area. The *k*/*S*_Bet_ values of BiOBr, Ag_0.05_-BiOBr, Ag_0.1_-BiOBr and Ag_0.2_-BiOBr were 1.6 × 10^−5^, 2.6 × 10^−4^, 0.0012 and 4.9 × 10^−7^ g∙m^−2^∙min^−1^, respectively, indicating that the intrinsic photocatalytic activity of Ag_0.1_-BiOBr was much higher than that of BiOBr. Therefore, Ag_0.1_-BiOBr nanoplates have the highest photocatalytic efficiency for BPA degradation under visible-light irradiation. Compared with the photodegradation efficiency of BPA over other photocatalysts (Table S3), Ag_0.1_-BiOBr, still, exhibited highly photocatalytic efficiency for BPA. 

On the basis of these results, the improved photocatalytic performance of the samples can be attributed to the enhanced response to visible light and improved carrier separation and transfer efficiency. 

Moreover, the stability of Ag_0.1_-BiOBr during the photocatalytic degradation of BPA under visible-light irradiation is shown in Figures S2 and S3. In XRD spectra (Figure S2), the diffraction peaks of the Ag_0.1_-BiOBr samples after photocatalytic degradation of BPA were, also, indexed to tetragonal BiOBr (JCPDS No. 09-0393), indicating the high stability of phase composition of the sample. All these peaks showed sharp patterns and no impurity peaks were noticeable, indicating the high crystallinity and purity of the samples. Moreover, the Ag_0.1_-BiOBr materials maintained a two-dimensional nanoplates structure after degradation process (Figure S3), indicating that the products possessed high stability, which cannot be destroyed through the catalytic degradation of organic pollutants. Additionally, the smoothness of Ag_0.1_-BiOBr planes after the degradation process indicates that there was still no Schottky junction and heterojunction formation on BiOBr surface, after the degradation process. The phase composition and morphology remain consistent before and after the reaction, indicating high stability of Ag_0.1_-BiOBr.

### 3.4. Mechanism of BPA Photocatalytic Degradation

**Figure 7:**
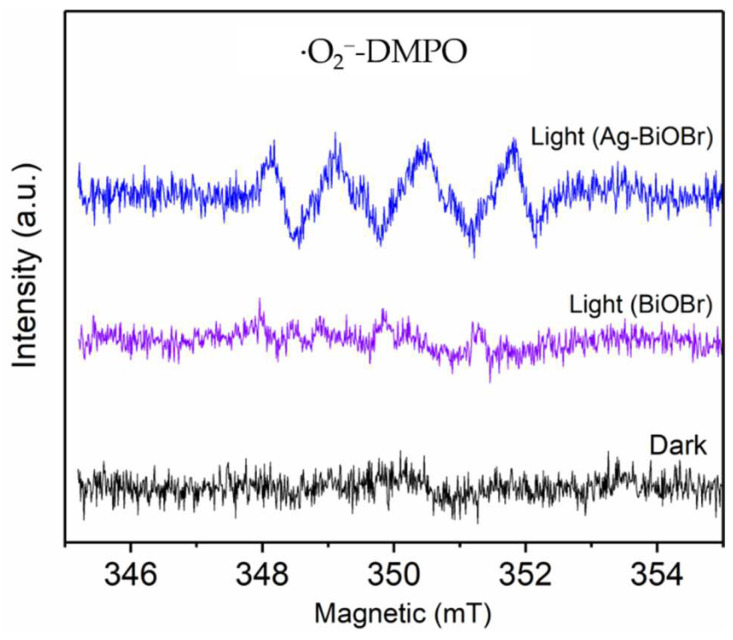
EPR spectra of BiOBr and Ag-BiOBr.

Reactive oxygen species (ROS) play an important role in photocatalytic process because of their strong oxidiazability and their ability to mineralize organic matter into carbon dioxide and water. EPR tests were carried out, to determine the existence of ROS and 5,5-dimethyl-1-pyrroline N-oxide (DMPO), as a spin-trap. As shown in Figure 7, no resonance signal is detected for the two samples in the dark. The ∙O_2_^−^ signal with a peak intensity of 1:1:1:1 were observed after 5 min irradiation [30,31,32]. In addition, the O_2_^−^ signal peak intensity of Ag_0.1_-BiOBr was much stronger that of BiOBr, demonstrating more generation of O_2_^−^, in the presence of Ag_0.1_-BiOBr. Moreover, to identify the contribution of different free radicals, free radical scavengers were added, separately, to remove different free radicals. Typically, Na_2_C_2_O_4_, ascorbic acid and t-butyl alcohol (TBA) were used to remove h^+^, O_2_^−^ and ∙OH, respectively. Compared with those without a free radical scavenger, the photocatalytic activity of Ag_0.1_-BiOBr were reduced by 9.76%, 78.05% and 24.39%, with the addition of Na_2_C_2_O_4_, ascorbic acid and TBA (Figure S4), respectively. The results show that the addition of ascorbic acid to remove ∙O_2_^−^ could significantly suppress the reaction, demonstrating that ∙O_2_^−^ was the main active species during the photocatalytic degradation process. 

**Figure 8:**
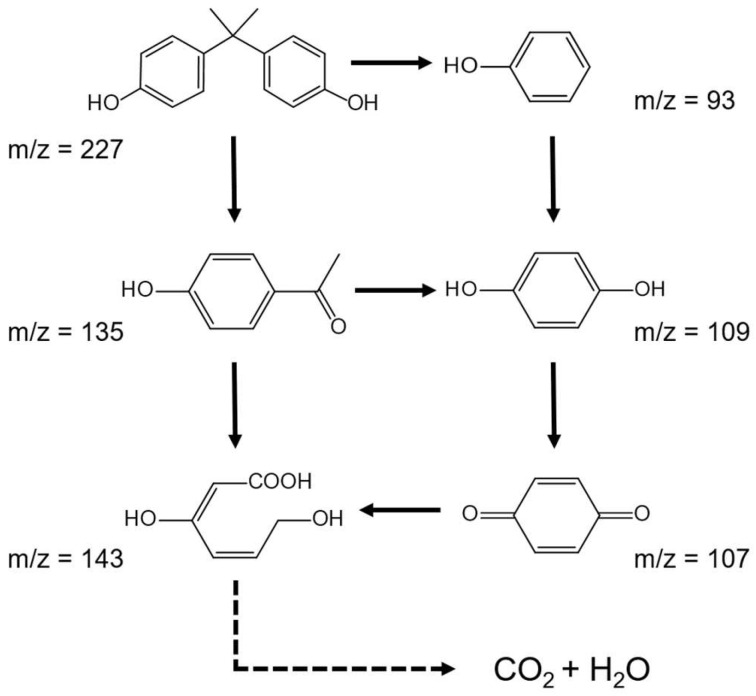
Proposed photocatalytic BPA degradation pathway.

Based on the identified intermediate compounds, the BPA degradation pathway was proposed in Figure 8. The two original intermediates, decomposed from BPA, with m/z levels that were 135 and 93, respectively, might be the first step of BPA degradation. Subsequently, the intermediates further turned into 1,4-benzoquinone (*m*/*z* = 109) and phydroxyacetophenone (*m*/*z* = 107). Then, the product ion at *m*/*z* 143 was generated, with a broken benzene-ring structure. Finally, these organic compounds would mineralize into CO_2_ and H_2_O.

## 4. Conclusions

Ag-doped BiOBr photocatalysts were prepared through hydrothermal procedure and characterized through a series of means. Ag-doping modification did not change the morphology and crystal structure but adjusted the band structure of BiOBr, and the band gap was reduced from 2.80 eV to 2.56 eV. Ag_0.1_-BiOBr exhibited the highest activity for the photocatalytic degradation of BPA, whose degradation pathway was proposed. Two aspects lead to the enhancement of the photocatalytic performance. The absorption edge of BiOBr redshifted from 453 nm to 462 nm, after Ag-doping modification, resulting in the enhanced visible-light-harvesting efficiency. Moreover, the high stability of the Ag-BiOBr nanosheets was, also, confirmed, which could be inferred from the consistency of morphology and structure before and after the reaction. Additionally, the improvement of carrier separation efficiency and photogenerated e^–^ reducibility could facilitate the generation of O_2_^−^, which was confirmed as the main active species for the degradation of BPA. 

## Data Availability

The data presented in this study are available on request from the corresponding author.

## Supplementary Materials

The following supporting information can be downloaded at: https://www.mdpi.com/article/10.3390/nano12111909/s1. Figure S1: XPS survey spectra of BiOBr and Ag-BiOBr; Figure S2: XRD spectra of Ag_0.1_-BiOBr before and after reaction; Figure S3: SEM images of Ag_0.1_-BiOBr, (a–c) before and (d,f) after reaction; Figure S4: kinetics constants of photocatalytic degradation of BPA over Ag_0.1_-BiOBr with and without added free radical scavengers; Table S1: comparison with the already published work; Table S2: BET surface areas, pore volume and pore size of the samples; Table S3: the comparison of photodegradation efficiency of BPA over different photocatalysts. References [33,34,35,36,37] are cited in the Supplementary Materials.

## References

[1] Morin-Crini N., Lichtfouse E., Fourmentin M., Ribeiro A.R.L., Noutsopoulos C., Mapelli F., Fenyvesi E., Vieira M.G.A., Picos-Corrales L.A., Moreno-Pirajan J.C. (2022). Removal of emerging contaminants from wastewater using advanced treatments. A review. Environ. Chem. Lett..

[2] Fujishima A., Honda K. (1972). Electrochemical photolysis of water at a semiconductor electrode. Nature.

[3] Chong M.N., Jin B., Chow C.W.K., Saint C. (2010). Recent developments in photocatalytic water treatment technology: A review. Water Res..

[4] Cheng H.F., Huang B.B., Dai Y. (2014). Engineering BiOX (X = Cl, Br, I) Nanostructures for highly efficient photocatalytic applications. Nanoscale.

[5] Wang Z.W., Chen M., Huang D.L., Zeng G.M., Xu P., Zhou C.Y., Lai C., Wang H., Cheng M., Wang W.J. (2019). Multiply structural optimized strategies for bismuth oxyhalide photocatalysis and their environmental application. Chem. Eng. J..

[6] Lei H., Zhang H.H., Zou Y., Dong X.P., Jia Y.M., Wang F.F. (2019). Synergetic photocatalysis/piezocatalysis of bismuth oxybromide for degradation of organic pollutants. J. Alloy. Compd..

[7] Qu S.Y., Xiong Y.H., Zhang J. (2018). Graphene oxide and carbon nanodots co-modified BiOBr nanocomposites with enhanced photocatalytic 4-chlorophenol degradation and mechanism insight. J. Colloid Interf. Sci..

[8] Ye L.Q., Su Y.R., Jin X.L., Xie H.Q., Zhang C. (2014). Recent advances in BiOX (X = Cl, Br and I) Photocatalysts: Synthesis, modification, facet effects and mechanisms. Environ. Sci. Nano.

[9] Wei X.X., Chen C.M., Guo S.Q., Guo F., Li X.M., Wang X.X., Cui H.T., Zhao L.F., Li W. (2014). Advanced visible-light-driven photocatalyst BiOBr-TiO_2_-graphene composite with graphene as a nano-filler. J. Mater. Chem. A.

[10] Wang C.Y., Zeng Q., Zhu G.C. (2021). Novel S-doped BiOBr nanosheets for the enhanced photocatalytic degradation of bisphenol a under visible light irradiation. Chemosphere.

[11] Huang W.M., Hua X., Zhao Y.P., Li K., Tang L.P., Zhou M., Cai Z.S. (2019). Enhancement of visible-light-driven photocatalytic performance of BiOBr nanosheets by Co^2+^ doping. J. Mater. Sci. Mater. Electron..

[12] Wu Z.H., Liu J., Tian Q.Y., Wu W. (2017). Efficient visible light formaldehyde oxidation with 2D *p-n* heterostructure of BiOBr/BiPO_4_ nanosheets at room temperature. ACS Sustain. Chem. Eng..

[13] Dong Y.M., Feng C.Y., Zhang J.J., Jiang P.P., Wang G.L., Wu X.M., Miao H.Y. (2015). A new p-metal-n structure AgBr-Ag-BiOBr with superior visible-light-responsive catalytic performance. Chem. Asian J..

[14] Liu Q., Yu Z.B., Li M.J., Hou Y.P., Sun L., Wang L., Peng Z.B., Chen D.M., Liu Y.X. (2017). Fabrication of Ag/AgBr/Ga_2_O_3_ Heterojunction Composite with Efficient Photocatalytic Activity. Mol. Catal..

[15] Javaid R., Qazi U.Y., Ikhlaq A., Zahid M., Alazmi A. (2021). Subcritical and supercritical water oxidation for dye decomposition. J. Environ. Manag..

[16] Javaid R., Qazi U.Y., Kawasaki S.I. (2016). Highly efficient decomposition of remazol Brilliant Blue R using tubular reactor coated with thin layer of PdO. J. Environ. Manag..

[17] Chen Q.L., Zhang Y.L., Zhang D.D., Yang Y.Q. (2017). Ag and N Co-doped TiO_2_ nanostructured photocatalyst for printing and dyeing wastewater. J. Water Process Eng..

[18] Zhai Y.J., Chen X.Y., Li J.H., Chu X.Y., Xu M.Z., Jin F.J., Li X., Fang X., Wei Z., Wang X.H. (2016). Preparation and properties of Ag doped Zno nanorods with N plasmon treatment. Ferroelectrics.

[19] Zhang W.D., Dong F., Xiong T., Zhang Q. (2014). Synthesis of BiOBr-graphene and BiOBr-graphene oxide nanocomposites with enhanced visible light photocatalytic performance. Ceram. Int..

[20] Phu N.D., Hoang L.H., Hai P.V., Huy T.Q., Chen X.B., Chou W.C. (2020). Photocatalytic activity enhancement of Bi_2_WO_6_ Nanoparticles by Ag doping and Ag nanoparticles modification. J. Alloy. Compd..

[21] Wu D., Ye L.Q., Yip H.Y., Wong P.K. (2017). Organic-free synthesis of {001} facet dominated BiOBr nanosheets for selective photoreduction of CO_2_ to CO. Catal. Sci. Technol..

[22] Wu X.Y., Zhang K.K., Zhang G.K., Yin S. (2017). Facile Preparation of BiOX (X = Cl, Br, I) nanoparticles and up-conversion phosphors/BiOBr composites for efficient degradation of NO gas: Oxygen vacancy effect and near infrared light responsive mechanism. Chem. Eng. J..

[23] Di J., Chen C., Zhu C., Song P., Xiong J., Ji M., Zhou J., Fu Q., Xu M., Hao W. (2019). Bismuth vacancy-tuned bismuth oxybromide ultrathin nanosheets toward photocatalytic CO_2_ reduction. ACS Appl. Mater. Inter..

[24] Cao D.L., Ma D.K., Zhou Z.L., Xu C.L., Cao C., Zhao P.Y., Huang Q.L. (2019). Efficient photocatalytic degradation of herbicide glyphosate in water by magnetically separable and recyclable BiOBr/Fe_3_O_4_ nanocomposites under visible light irradiation. Chem. Eng. J..

[25] Patil S.S., Mali M.G., Hassan M.A., Patil D.R., Kolekar S.S., Ryu S.W. (2017). One-pot in situ hydrothermal growth of BiVO_4_/Ag/rGo hybrid architectures for solar water splitting and environmental remediation. Sci. Rep..

[26] Baby B.H., Thomas A.M., Amrutha E.G., Mohan D.B. (2020). Enhancement of optoelectronic properties via substitutional doping of Cu, in and Ag in SnS nanorods for thin film photovoltaics. Sol. Energy.

[27] Patil S.S., Mali M.G., Tamboli M.S., Patil D.R., Kulkarni M.V., Yoon H., Kim H., Al-Deyab S.S., Yoon S.S., Kolekar S.S. (2016). Green approach for hierarchical nanostructured Ag-Zno and their photocatalytic performance under sunlight. Catal. Today.

[28] Meng X.C., Jiang L.Y., Wang W.W., Zhang Z.S. (2015). Enhanced photocatalytic activity of BiOBr/ZnO heterojunction semiconductors prepared by facile hydrothermal method. Int. J. Photoenergy.

[29] Shang J., Hao W.C., Lv X.J., Wang T.M., Wang X.L., Du Y., Dou S.X., Xie T.F., Wang D.J., Wang J.O. (2014). Bismuth oxybromide with reasonable photocatalytic reduction activity under visible light. ACS Catal..

[30] Khachatryan L., Vejerano E., Lomnicki S., Dellinger B. (2011). Environmentally persistent free radicals (EPFRs). 1. generation of reactive oxygen species in aqueous solutions. Environ. Sci. Technol..

[31] Zeng Q., Wang C.Y., Xu B.X., Han J.Y., Fang X., Zhu G.C. (2022). Electron-level mechanistic insights into Ce doping for enhanced efficiency degradation of bisphenol a under visible light irradiation. Nanomaterials.

[32] Wu D., Yue S.T., Wang W., An T.C., Li G.Y., Yip H.Y., Zhao H.J., Wong P.K. (2016). Boron doped BiOBr nanosheets with enhanced photocatalytic inactivation of Escherichia coli. Appl. Catal. B Environ..

[33] Liu T., Zhang Y., Shi Z., Cao W., Zhang L., Liu J., Chen Z. (2021). BiOBr/Ag/AgBr heterojunctions decorated carbon fiber cloth with broad-spectral photoresponse as filter-membrane-shaped photocatalyst for the efficient purification of flowing wastewater. J. Colloid Inter. Sci..

[34] Carolina O.N., Yuri P., Varsha S., Netzahualpille H., Jürgen M., Mika S., Nancy O.S. (2021). Gd^3+^ doped BiVO_4_ and visible light-emitting diodes (LED) for photocatalytic decomposition of bisphenol A, bisphenol S and bisphenol AF in water. J. Environ. Chem. Eng..

[35] Yuan T., Xiaohong Y., Manman M., Yue J., Xiaoli L., Hao Z., Tianwei O. (2020). Anatase TiO_2_@MIL-101(Cr) nanocomposite for photocatalytic degradation of bisphenol A. Colloid Surface A.

[36] Ye C., Hu K., Niu Z., Lu Y., Zhang L., Yan K. (2019). Controllable synthesis of rhombohedral α-Fe_2_O_3_ efficient for photocatalytic degradation of bisphenol A. J. Water Process Eng..

[37] Wang C.Y., Xing Z., Qiu H.B., Wang W.K., Huang G.X., Jiang J., Yu H.Q. (2017). Photocatalytic degradation of bisphenol A by oxygen-rich and highly visible-light responsive Bi_12_O_17_Cl_2_ nanobelts. Appl. Catal. B Environ..

