# Supplementary material for: Visible-Light-Driven Ag-Doped BiOBr Nanoplates with an Enhanced Photocatalytic Performance for the Degradation of Bisphenol A
Source: Nanomaterials (Basel). 2022 Jun 2;12(11):1909. doi: 10.3390/nano12111909 (PMC9182274; doi:10.3390/nano12111909)
Supplement: Supplementary file 1 [file nanomaterials-12-01909-s001.zip › nanomaterials-1719640-supplementary.pdf]

nanomaterials

Supplementary Material

Visible-Light-Driven Ag-Doped BiOBr Nanoplates with an Enhanced Photocatalytic Performance for the Degradation of Bisphenol A

Chu-Ya Wang *, Qi Zeng, Li-Xia Wang, Xin Fang and Guangcan Zhu *

School of Energy and Environment, Southeast University, Nanjing 210096, China; zeng_qi@seu.edu.cn (Q.Z.); 220210571@seu.edu.cn (L.-X.W.); fxin@seu.edu.cn (X.F.)

* Correspondence: wang-cy@seu.edu.cn (C.-Y.W.); gc-zhu@seu.edu.cn (G.Z.); Tel.: +86-177-7534-6164 (C.-Y.W.); +86-189-1296-6396 (G.Z.)

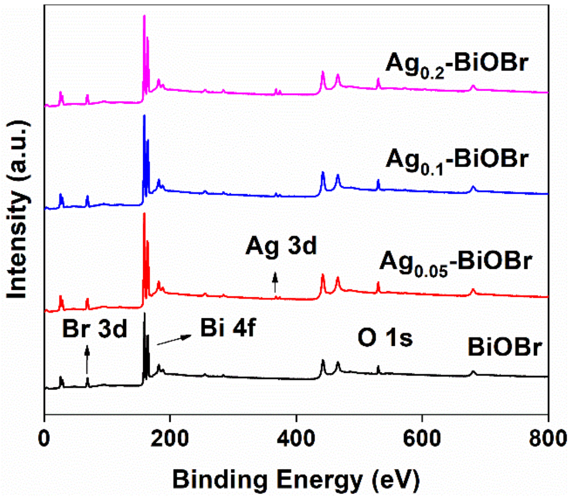

Figure S1. XPS survey spectra of BiOBr and Ag-BiOBr.

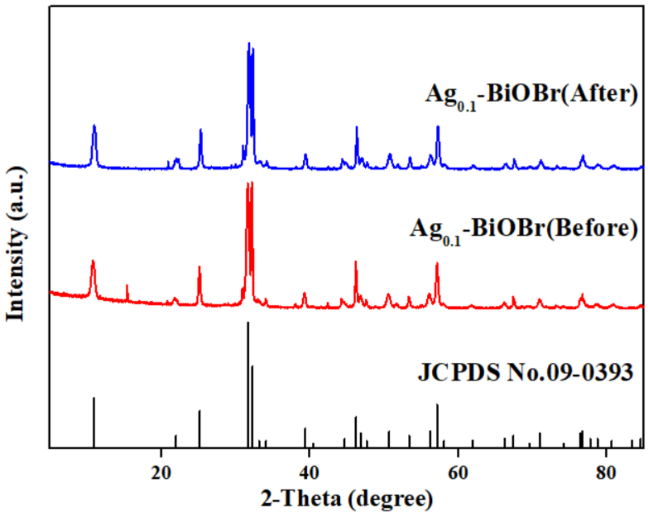

Figure S2. XRD spectra of $Ag_{0.1}$-BiOBr before and after reaction.

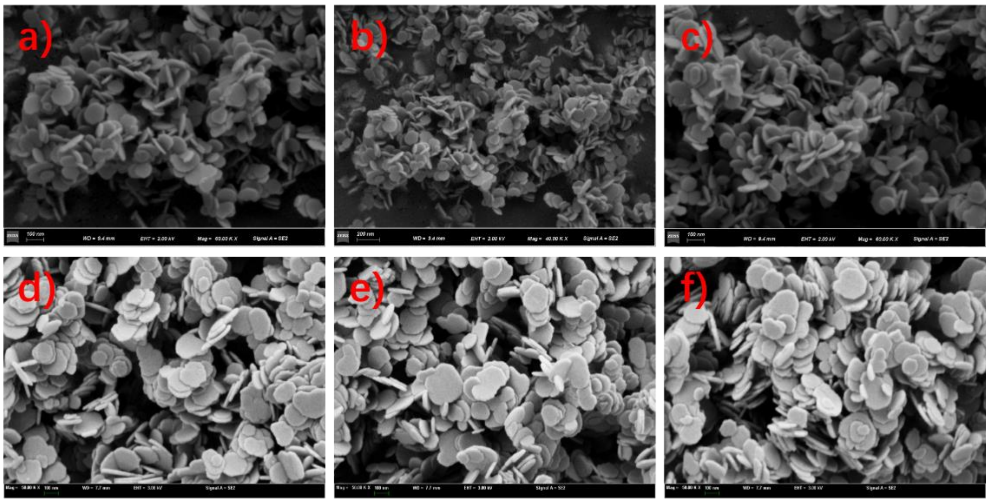

Figure S3. SEM images of $Ag_{0.1}$-BiOBr (a-c) before and (d-f) after reaction.

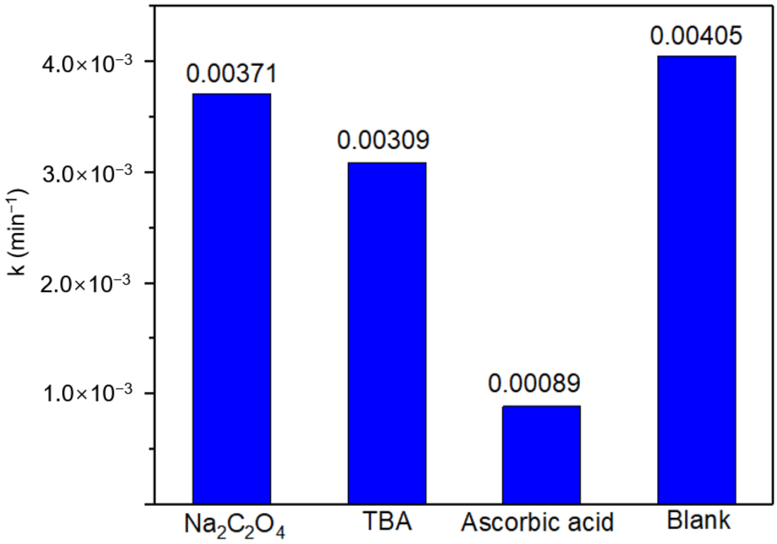

Figure S4. Kinetics constants of photocatalytic degradation of BPA over $Ag_{0.1}$-BiOBr with and without added free radical scavengers.

Table S1. Comparison with the already published work.

Previous work	In our work	Ref.
• The combination of AgBr-Ag-BiOBr possessed a p-metal-n structure.<br>• The combination of Ag/AgBr/BiOBr was a Z-scheme heterojunction.<br>• The target pollutants were methyl orange, which can induce dye sensitization.<br>• The target pollutants were RhB and acid orange 7 with color which can induce dye sensitization.	• The pollutant used herein was BPA which is a kind of colorless endocrine disrupting compound (EDC) without dye sensitization.<br>• The characterizations of products demonstrated that BiOBr possessed a smooth surface, indicating that there was no heterojunction or Schottky junction. Ag elements displayed a high dispersion in BiOBr crystal with the form of Ag(I) instead of Ag(0).	[1-2]

• The light source is UV light.<br>• The target pollutant was methyl orange (MO), a colored dye, could induce dye sensitization.	• In this work, we used 420 nm cutoff filter to simulate the visible light illumination condition.<br>• The pollutant used herein was BPA which is a kind of colorless edcs without dye sensitization	[3]

Table S2. BET surface areas, pore volume and pore size of the samples.

Samples	BET surface area (m^2/g)	Pore volume (cm^3/g)	Pore size (nm)
BiOBr	12.2749	0.043885	14.30080
$Ag_{0.05}$-BiOBr	15.4384	0.107037	27.73252
$Ag_{0.1}$-BiOBr	15.2018	0.102173	26.88460
$Ag_{0.2}$-BiOBr	20.5491	0.185371	36.08350

Table S3. The comparison of photodegradation efficiency of BPA over different photocatalysts.

Photocatalysts	Source of light	Amount of catalyst	Initial concentration of BPA	Amount of BPA solution	Reaction time	Degradation rate	Ref.
		(mg)	(mg/L)	(mL)	(min)		
Gd^{3+} doped $BiVO_4$	LED lamp (36 W, 730 lm)	1000	10	<50	180	80%	[4]
TiO_2@MIL-101(Cr)	125 W mercury lamp	40	50	80	240	92%	[5]
α-Fe_2O_3	Visible light (high pressure 300W Xenon lamp)	50	——	50	360	93%	[6]
$Bi_{12}O_{17}Cl_2$	Visible light (>420 nm, 500W Xenon lamp)	20	10	40	120	55%	[7]
$Ag_{0.1}$-BiOBr	Visible light (>420 nm, 500W Xenon lamp)	10	10	50	140	82%	This work

References

1. Dong, Y.; Feng, C.; Zhang, J.; Jiang P.; Wang, G.; Wu, X.; Miao. A new p-metal-n structure AgBr-Ag-BiOBr with superior visible-light-responsive catalytic performance. *Chem. Asian J.* **2015**, *10*, 687-93.
2. Liu, T.; Zhang, Y.; Shi, Z.; Cao, W.; Zhang, L.; Liu, J.; Chen, Z. BiOBr/Ag/AgBr heterojunctions decorated carbon fiber cloth with broad-spectral photoresponse as filter-membrane-shaped photocatalyst for the efficient purification of flowing wastewater. *J. Colloid Inter. Sci.* **2021**, *587*, 633-643.
3. Liu, Q.; Yu, Z.; Li, M.; Hou, Y.; Sun, L.; Wang, L.; Peng, Z.; Chen, D.; Liu, Y. Fabrication of Ag/AgBr/Ga_2O_3 heterojunction composite with efficient photocatalytic activity. *Mol. Catal.* **2017**, *432*, 57-63.
4. Carolina, O.N.; Yuri, P.; Varsha, S.; Netzahualpille, H.; Jürgen, M.; Mika, S. and Nancy, O.S. Gd^{3+} doped $BiVO_4$ and visible light-emitting diodes (LED) for photocatalytic decomposition of bisphenol A, bisphenol S and bisphenol AF in water. *J. Environ. Chem. Eng.* **2021**, *9*, 105842.
5. Yuan, T.; Xiaohong, Y.; Manman, M.; Yue, J.; Xiaoli, L.; Hao, Z.; Tianwei, O. Anatase TiO_2@MIL-101(Cr) nanocomposite for photocatalytic degradation of bisphenol A. *Colloid Surface A* **2020**, *596*, 124745.
6. Ye, C.; Hu, K.; Niu, Z.; Lu, Y.; Zhang, L. and Yan, K. Controllable synthesis of rhombohedral α-Fe_2O_3 efficient for photocatalytic degradation of bisphenol A. *J. Water Process Eng.* **2019**. *27*, 205-210.
7. Wang, C.Y.; Xing, Z.; Qiu, H.B.; Wang, W.K.; Huang, G.X.; Jiang, J. and Yu, H.Q. Photocatalytic degradation of bisphenol A by oxygen-rich and highly visible-light responsive $Bi_{12}O_{17}Cl_2$ nanobelts. *Appl. Catal. B: Environ.* **2017**, *200*, 659-665.
